# Assessing the nutritional content and adequacy of food parcels among vulnerable Lebanese during a double crisis: COVID-19 pandemic and an economic meltdown

**DOI:** 10.1017/S1368980023000241

**Published:** 2023-06

**Authors:** Lama Mattar, Hussein Hassan, Nour Kalash, Dana Malli, Marwa Diab-El-Harake, Sahar Nassour, Lamis Jomaa

**Affiliations:** 1 Nutrition Program, Department of Natural Sciences, School of Arts and Sciences, Lebanese American University, Beirut, Lebanon; 2 Department of Nutrition and Food Sciences, Faculty of Agricultural and Food Sciences, American University of Beirut, Beirut, Lebanon; 3 Department of Human Sciences, College of Health and Sciences, North Carolina Central University, Durham, NC, USA; 4 Faculty of Agricultural and Food Sciences, American University of Beirut, Beirut, Lebanon

**Keywords:** Food parcel, Food security, Nutrition guidelines, Economic crisis, COVID-19, Lebanon

## Abstract

**Objectives:**

This study aimed to explore the nutritional content and quality of food parcels distributed in Lebanon and assess their adherence to dietary guidelines during the COVID-19 pandemic and an unprecedented economic crisis.

**Design:**

Cross-sectional study (June–July 2020); phone survey (thirty items).

**Setting:**

Lebanon.

**Participants:**

Food parcel providers (FPP; *n* 72) involved in food parcel distribution (FPD), mainly to Lebanese households.

**Results:**

FPP included international non-governmental organizations (INGO) (*n* 3), local non-governmental organizations (*n* 45) and personal initiatives (*n* 24). Overall, low adherence to the World Food Programme (WFP) food parcel guidelines were observed among FPP for specific food items, including vegetables, fish, legumes and cereals, whereas salt content significantly surpassed the guidelines (all *P*-values <0·001). On average, a food parcel provided 608·4 ± 55 kcal/d/person. The greatest contributors to total energy intake (TE) in the food parcel were carbohydrates (46·4 %) and fats (46·8 %), while protein contributed to 7 %TE. In addition, %TE from fats and sugars significantly surpassed the dietary reference intakes (DRI) for a single person per d (134–234 % and 185 % of DRI, respectively, *P*-values <0·001). Only 10–15 % of daily needs for key micronutrients, including Fe, Zn, thiamin, riboflavin and dietary folate, were met through the food parcels. Adequate food safety and hygiene practices were reported among FPP, yet dramatic changes in food costs due to overlapping crises affected the quality and quantity of food in parcels.

**Conclusions:**

Findings highlight the need to improve the nutritional content of food parcels and adherence to dietary guidelines to alleviate food and nutrition insecurity while preventing diet-related diseases among vulnerable beneficiaries in Lebanon.

Food insecurity has been on the rise throughout the world since 2020, especially in low- and middle-income countries, as a result of the COVID-19 pandemic and its economic repercussions^(1)^. Amidst the pandemic, food aid was one of the key operations that provided millions of vulnerable households and individuals worldwide with access to food. The positive impact of food aid, including food parcel distribution (FPD) in alleviating food insecurity, has been well documented in relief and development operations^(2,3)^. However, the impact of food aid on dietary diversity and nutritional adequacy may differ depending on the modes of operations, nature and length of crises, adherence to nutritional guidelines among other factors^(4)^. The variability in modes of operations, purchasing capacities, distribution mechanisms and channels, resources and outreach aptitudes, as well as the adherence, or lack of, to international and local dietary guidelines and recommendations, can all affect the quality and quantity of food reaching the end users (beneficiaries) in the community^(5)^. Thus, there is a need to further explore the nutritional content and adequacy of food parcels, as an element affecting community’s health and well-being.

Lebanon represents a unique setting to explore the nutritional adequacy of food parcels amidst multiple crises, including the pandemic and a concurrent economic recession, that further exacerbated poverty and food insecurity across its population^(6)^. A small country on the Eastern coast of the Mediterranean, Lebanon has undergone decades of political, economic and social turmoil that weakened the healthcare system, depleted the country’s resources and diminished its welfare and social safety net programmes^(7)^. In parallel, the country continues to have the highest concentration of refugees per capita further straining its limited resources, despite one of the largest humanitarian operations in the region in response to the Syrian crisis^(8)^. Additionally, Lebanon has a long-standing high dependence on food imports (65–85 % of food basket is imported including staple foods such as wheat) and has limited agricultural productivity and capacity^(9)^. Thus, food insecurity has been a serious concern for Lebanese households and displaced population groups, even prior to the pandemic with rates reaching 49 % among Lebanese households with children (4–18 years old) in 2015^(10)^, and even higher levels of food insecurity reported among displaced Syrian, Palestinian and Iraqi refugees^(7,11–13)^. With the start of the national protests in October 2019, and the emergence of the COVID-19 pandemic, Lebanon has been in the eye of the storm witnessing overlapping crises, including a political uprising, a health pandemic with repetitive lockdown measures and an unprecedented economic free fall. The financial crisis marked by strict capital controls, exhaustion of central bank reserves and a stringent currency devaluation pushed people further into poverty (55 %) and unemployment (reaching about 40 % by 2020)^(14)^. Food prices has also risen drastically since the start of the financial crisis and were further exacerbated by the pandemic^(15,16)^. Recent estimates of food insecurity in the country show that more than 57 % of the Lebanese families are facing severe economic challenges to access food^(17)^.

Since the start of the Syrian war in 2011 and the large displacement of refugees, Lebanon has been also home to a large humanitarian operation with well-developed and coordinated mechanisms for food and basic assistance distribution provided by UN agencies and their international and local partners. These programmes have been targeting the most vulnerable population groups, including Syrian and Palestinian refugees and Lebanese host communities^(18)^. However, the multiple crises witnessed in Lebanon since 2019, along with the worsening food security situation, have led to uncoordinated FPD mechanisms. Community-based organisations, including local charities, non-governmental organisations (NGO), religious groups and personal initiatives, were increasingly involved in FPD to help address the growing needs of the Lebanese population, yet the food relief efforts of these entities were not always integrated into the national-level frameworks of food assistance. In addition, limited data existed as to the level of readiness of such organisations to adopt existing operational guidance, protocols and procedures that are enforced by the international humanitarian agencies and their partners who have been working with the Syrian refugee population present in the county for more than a decade^(19,20)^. In addition, to our knowledge, there remains a lack of in-depth assessment of the nutritional content and adequacy of food parcels distributed by community-based organisations and initiatives in meeting the dietary needs of the beneficiaries. Consequently, the present study aims to address this gap through: first, assessing and characterising the FPD led by community organisations and charity groups across Lebanon during the double crises; second, comparing the level of adherence of FPP and their food parcels to the locally tailored WFP guidelines for food basket composition and to the minimal safety standards; and third, evaluating the nutritional quality and adequacy of the food parcels distributed to beneficiaries in the country.

## Methods

### Study design and protocol

A cross-sectional observational design was adopted in the present study. Data were collected via a semi-structured phone survey with representatives from local and international non-governmental organisations (INGO), community groups and charities involved in FPD in Lebanon. A convenience sampling approach was adopted as part of the present study. First, a database of food parcel providers (FPP) (i.e. INGO/local NGO, individual initiatives, community groups, religious institutions and charity associations) distributing food aid to households across Lebanon was consolidated by the research team, through multiple venues including the database of the Lebanon Crisis Response Plan (LCRP) – Food Security Sector (FSS), online search, social media surveying and referrals. Inclusion criteria for FPP in the present study were (i) organisations that are involved in the distribution of dry food rations/food parcels and (ii) organisations that serve primarily Lebanese households in various governorates across the country (Beirut, Mount Lebanon, Bekaa, Baalbeck-Hermel, North, Akkar, South and Nabatieh). Exclusion criteria were (i) organisations not serving dry rations and (ii) INGO or local organisations working primarily with non-Lebanese households, including Syrian and Palestinian refugee populations.

### Data collection

Data collection spanned between June and July 2020 and ended 2 weeks prior to the tragic Beirut port blast that took place on 4 August, 2020. Phone interviews were conducted by trained research team members using a thirty-item questionnaire. The survey was developed by the research team while adapting themes raised in previous similar surveys from similar food insecurity contexts^(21–23)^. Briefly, questions included general characteristics of the FPP, regions where food distribution is taking place, selection criteria for beneficiaries, content and type of foods (in quantity and quality) within the food parcels, and food parcel prices and food safety measures adopted as part of the FPD. In addition, open-ended questions were included to further consider the challenges confronted by the FPP and their interest in improving their food aid process, followed by food parcel-specific questions. A list or a photo of the items placed in the food parcels were requested to be sent along with the quantities for each item. Regarding food safety questions, and in the case where the FPP was receiving sealed and ready-made parcels from a third party, ‘NA’ (not applicable) was noted. Weights of items were stated in kilograms (kg), whereas for items such as cheese and tea, quantities were noted in pack or box. After data entry, all food items variables with a frequency of 10 and lower were included under ‘Other food items’ instead of separate variables. Food items were divided into seven categories based on the WFP locally developed and tailored guidelines on food parcels composition (cereals, legumes, fish, oil, sugar, salt and vegetables)^(24)^. Each interview took on average 20 min. Interviews were conducted in either English or Arabic language depending on the preference of the FPP representative/contact person.

### Adherence to dietary guidelines and nutrient analysis of food parcels

Content of each food parcel was recorded by the research team members (data collectors), and the specified amount of food items (in kg, liters, packs, boxes, etc.) were entered into an SPSS spreadsheet. The food parcel was per household; however, the number of household members receiving the food parcel by FPP was also recorded. Next, the adherence of the FPP and their respective food parcels to the locally tailored WFP guidelines for food parcel composition was assessed^(26)^. The total amount of food items provided per parcel per household (e.g. in kg, l) was compared to the recommended amount of food groups (in weight) as well as in terms of percentage adherence of FPP to the recommended amounts for each of the food items (i.e. % FPP adhering to the recommended amounts of food items per parcel, as per the locally tailored WFP guidelines); see online Supplemental material (Table S1).

To assess the nutritional adequacy of the food parcels provided by FFP, the contents of food parcels were standardised to a single person per d to be able to compare the contents of the different types of food parcels. The provided amount of food items (in kg) was first converted to grams (g). Second, the amount of food (in g) was divided by the total number of household members to obtain individual intake. Since FPP provided food parcels that last 1 month, food amounts were then divided by 30 d to estimate daily individual food intakes. Next, these individual or single person estimated food intakes were entered into Nutritionist Pro Software (Nutritionist Pro, Axxya Systems, San Bruno, CA, USA, version 5.1.0, 2018) to estimate energy, macro- and micronutrient intakes per person per d from each of these FPP food parcels. Within the Nutritionist Pro, the USDA database was selected for analysis, as it includes a wide range of international brands and commercial products available in the Lebanese market. In addition, local food products and food items were added to the database using available food composition databases and labels^(24,25)^. Given there are no gender- or age-specific dietary reference intakes (DRI) for Lebanese or Middle Eastern populations, the derived energy and nutrient values were then compared with the US-based DRI for adults, as per the Institute of Medicine^(26,27)^ and to the international WFP/WHO/FAO guidelines^(28)^. Absolute values (g, mg and mcg) were presented as mean values and standard errors, and relative values, such as percent of total energy (TE) from macronutrients, were presented as percentages of total energy (% TE).

### Statistical analysis

Survey data were analysed using SPSS software (version 26). Descriptive statistics were used to summarise the characteristics of FPP and beneficiaries. Continuous variables were presented as mean values and standard deviations (sd), whereas for dietary data, mean values with standard errors were reported. Categorical variables were presented as frequencies and proportions. Comparisons of mean-adjusted quantities for each food item (in kg) to the recommended WFP Food Basket Guidelines (monthly ration in kg) were assessed using a one-sample *t* test. Furthermore, adjusted quantities of food items in the food parcel were converted to percent adherence to the proposed WFP guidelines^(24)^. The level of adherence of the seven food items (mean percent, %) was further demonstrated for all FPP. In addition, percentage needs met for mean energy and nutrient content of food parcels (for a single person for 1 d) compared to the US DRI were computed. To assess whether the energy and nutrient content of a food parcel differed significantly from the dietary guidelines, a one-sample *t* test was used. Comparisons of prices of food parcels before and after the economic crisis (in US dollars and in Lebanese pounds) were conducted using paired *t* test. Statistical significance was set at a *P*-value lower than 0·05.

## Results

**Fig. 1:**
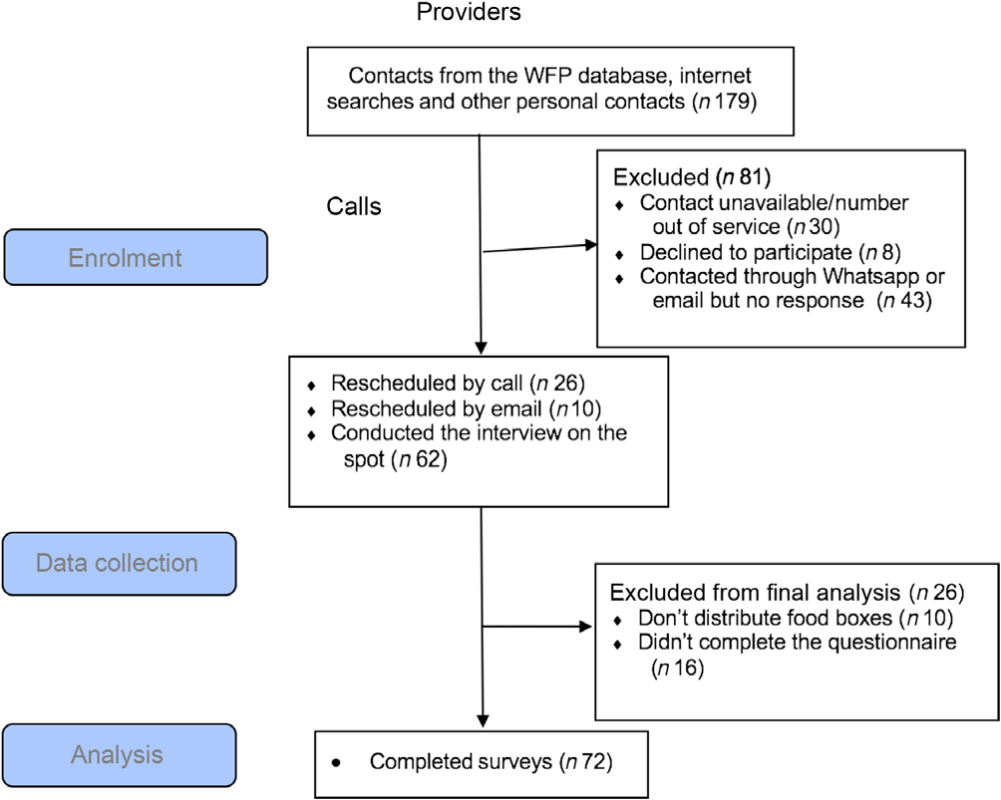
Flow diagram of the selection and data collection of food parcel. WFP, World Food Programme

A total of 179 FPP were identified from the initial search, yet eighty-one FPP were either not reachable (by phone calls/WhatsApp messages) or refused to participate in the study. The remaining ninety-eight FPP who expressed interest in the study were further screened and ten FPP were excluded from the study as they did not meet the inclusion criteria. An additional sixteen FPP did not have a complete questionnaire; thus, the final sample size included in the analysis was seventy-two FPP (Fig. 1).

**Fig. 2:**
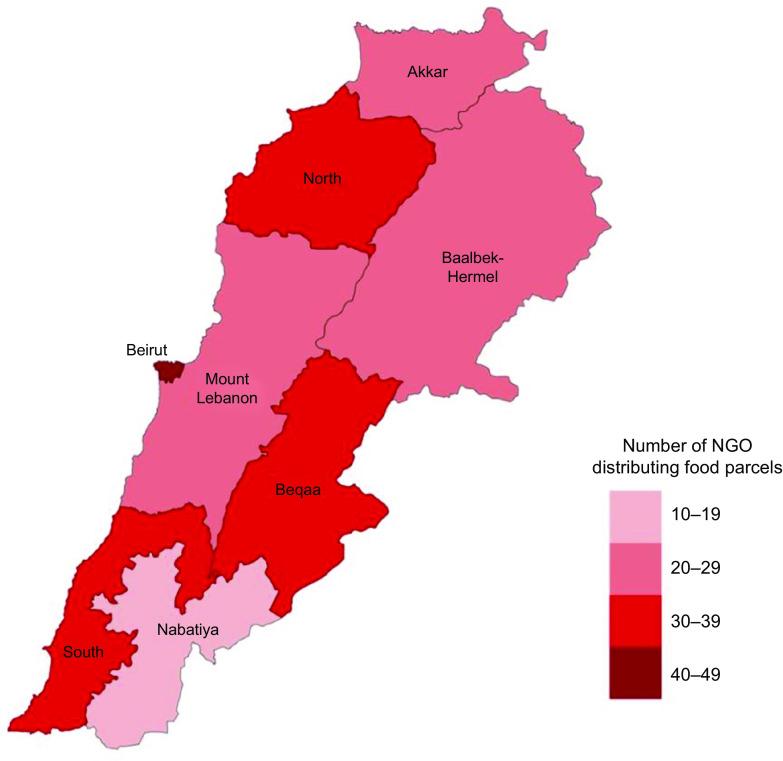
Number of FPP distributing food boxes to Lebanese households by governorate. FPP, food parcel providers. NGO, non-governmental organizations

From the seventy-two FPP that agreed to participate in the study and completed the phone interview, 63·9 % (*n* 46) had started FPD prior to the COVID-19 pandemic and the country’s first lockdown (March 2020), while the remaining 36·1 % (*n* 26) initiated their FPD during the pandemic. The FPP included in our study covered all eight governorates in Lebanon with the highest concentration of FPD by FPP included in our sample being reported in the capital Beirut (*n* 43) followed by the South (*n* 39), Beqaa (*n* 34), North Lebanon-(*n* 32), Mount Lebanon (*n* 29), Akkar (*n* 22), Baalbek-Hermel (*n* 21) and Nabatieh (*n* 19). Worth noting that each FPP reached beneficiaries in different governorates (on average 3·32 ± 2·65). The allocation density of FPP in the study sample is presented in Fig. 2.

### Characteristics of food parcel providers, beneficiaries receiving food parcels and logistics of food parcel distribution in the study sample

As seen in Table 1, in terms of the FPP characteristics, three FPP (4·2 %) were INGO (not including UN agencies), forty-five (62·5 %) FPP were local NGO and one-third of the FPP (*n* 24) were personal initiatives. Besides the FPD, 26·4 % of FPP were also distributing medical kits, 36·1 % were providing hygiene kits and 23·6 % were providing families with a hot meal alongside the dry rations/food parcels. The majority of FPP (73·6 %) had ongoing FPD at the time of data collection, and the rest were involved for shorter periods varying from a one-time distribution up to 6 months.

**Table 1:** Characteristics of the food parcel providers (FPP), beneficiaries receiving food aid and logistics of the food parcel distribution (FPD)

Variable	Total sample (*n* 72)	
	*n*	%
Characteristics of FPP		
FPP type		
International NGO*	3	4·2
Local NGO†	45	62·5
Personal initiatives	24	33·3
Other type of assistance provided by the FPP (more than one apply)		
Medical kits	19	26·4
Hygiene kits	26	36·1
Hot meals	17	23·6
Other assistance‡	36	50
Initiated FPD		
Before COVID-19	46	63·9
During COVID-19	26	36·1
Duration of FPD		
<2 weeks duration	2	2·8
2 weeks–1 month	5	7
1–3 months	7	9·7
>3 months	5	6·9
Ongoing	53	73·6
Source of funds for FPD (more than one apply)		
Internal funds	61	85·9
Donors	38	53·5
Fundraising events	21	29·6
Beneficiaries characteristics		
Beneficiary nationality (more than one apply)		
Lebanese	69	95·8
Syrian	37	51·4
Palestinian	27	37·5
Other nationalities§	67	93·1
Household selection criteria (more than one apply)		
Most needy	50	69·4
Without job	36	50
Without income	30	41·7
Presence of kids or elderly	21	29·2
Sickness	20	27·8
Number of individuals per house	20	27·8
Data list from NGO	19	26·4
Social criteria	14	19·4
Refugee camp	8	11·1
Randomly	6	8·6
Referrals	2	2·8
Type of assistance provided by other sources to beneficiaries		
Food parcel and/or hot meal (from other sources)	41	59·4
Cash/voucher	6	8·7
All of the above	3	4·4
No other sources	19	27·5
Criteria and guidelines adopted by FPP		
Criteria for selection of food items in the parcels (more than one apply)		
Necessities	36	52·2
Price	28	40·6
Quality	26	37·7
Availability of food items	25	36·2
Donations	22	31·9
Guidelines	21	31·3
Nutrition expert	6	8·7
Did FPP follow any nutrition guidelines in the selection of food items in their parcels?		
Yes	21	31·3
No	46	68·7
Did FPP participate in any trainings on FPD?		
Yes	17	23·9
No	54	76·1
Did FPP face any challenges during FPD?		
Yes	61	87·1
No	9	12·9
Type(s) of challenges faced by FPP (more than one apply)		
Logistics	32	46·4
Increased demand	30	42·9
Increased prices	28	40
COVID-19 lockdown	18	25·7
Reaching needy	14	20
Areas not applying COVID-19 safety precautions	13	18·6
Political	5	7·2
Accessibility/location	3	4·3
Donations	1	1·4

* International NGO excluded UN agencies and international partners involved in the distribution of food and humanitarian assistance primarily to Syrian refugees.

† Local NGO included religious and political groups involved in FPD.

‡ Other assistance includes clothes, cash, house rental, hospitalisation, school/college tuition and vouchers.

§ Other nationalities include families from Iraq, Ethiopia and Bangladesh.

In terms of beneficiaries’ characteristics, the majority of FPP (95·8 %) reported targeting Lebanese households and 51·4 % FPP were also serving Syrian refugee households. In addition, 26·4 % of the FPP were following data lists provided by other partners and NGO when selecting their beneficiaries, while other FPP were selecting beneficiaries based on their own selection criteria. In addition, slightly less than one-third of the interviewed FPP reported that the beneficiaries were not receiving any form of food aid, while the remaining 72 % of FPP representatives reported that beneficiaries are receiving either complementary food parcels and hot meals, cash/voucher assistance, or a combination of food and cash assistance (see Table 1).

In terms of the criteria and guidelines adopted by FPP in their food parcel preparation and distribution, on average, each food parcel distributed by the FPP covered five individuals per household for a period of 22·6 ± 10·8 d. Criteria for selection of food items in the parcel included items considered as necessities, availability and the price of the available items which was constantly changing, and the quality of the available food items.

### Food parcel content description

**Photo 1:**
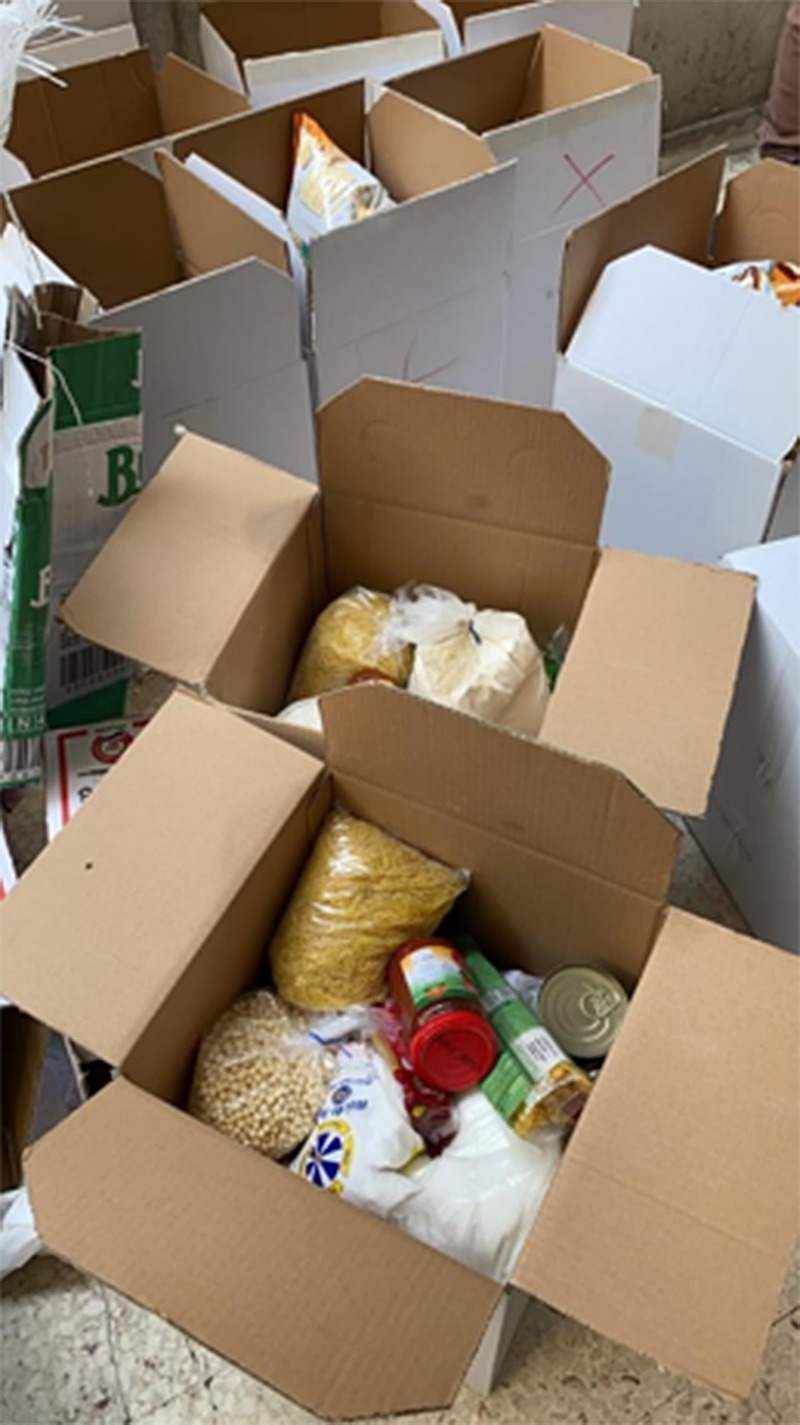
Samples of food parcels from FPP

**Photo 2:**
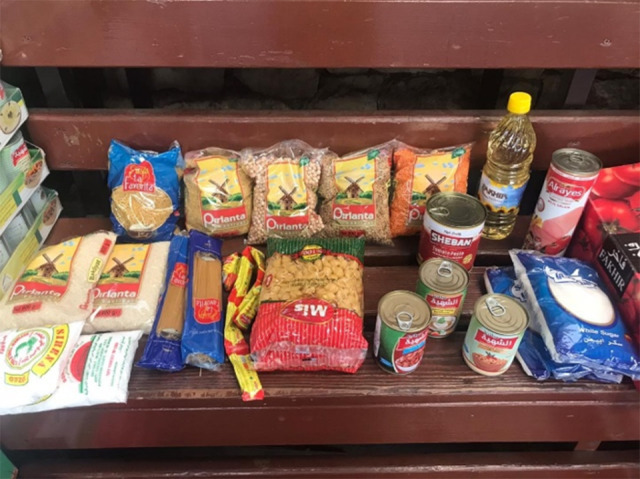
Sample of food parcel content

There was heterogeneity in the content of the food parcels as reported by the FPP. Generally, all food parcels included rice, and the majority included pasta, vermicelli, burghul (cracked parboiled groats), flour, lentils, beans and peas, oils, sugar and salt. Additionally, some food boxes included fish and meat products such as tuna, sardines, mortadella, and sweets such as jam and ‘halawi’ (cf. photos 1, 2).

Our results showed that almost 40 % of FPP (*n* 24) were distributing milk products as part of their food parcels, whereas the remaining 60 % FPP did not include any milk products. Of the twenty-four FPP who included milk products within the distributed food parcels, twenty-two confirmed distributing milk powder products and two FPP were distributing liquid milk.

### Level of adherence to nutrition guidelines

As presented in Table 1, overall, twenty-one of the sixty-seven FPP who responded to this question (31·3 %) reported that they followed any form of nutritional guidelines when planning and preparing the content of the food parcels. Guidelines referred to by the FPP were primarily the WFP food parcel guidelines with four organisations referring to other guidelines including the Ministry of Public Health and WHO guidelines. Other guidelines referred to were related to the food safety measures adopted during the COVID-19 pandemic. Among the fifteen organisations that reported following the WFP guidelines for food parcels, one was an INGO, twelve were local FPP and two were personal initiatives.

**Fig. 3:**
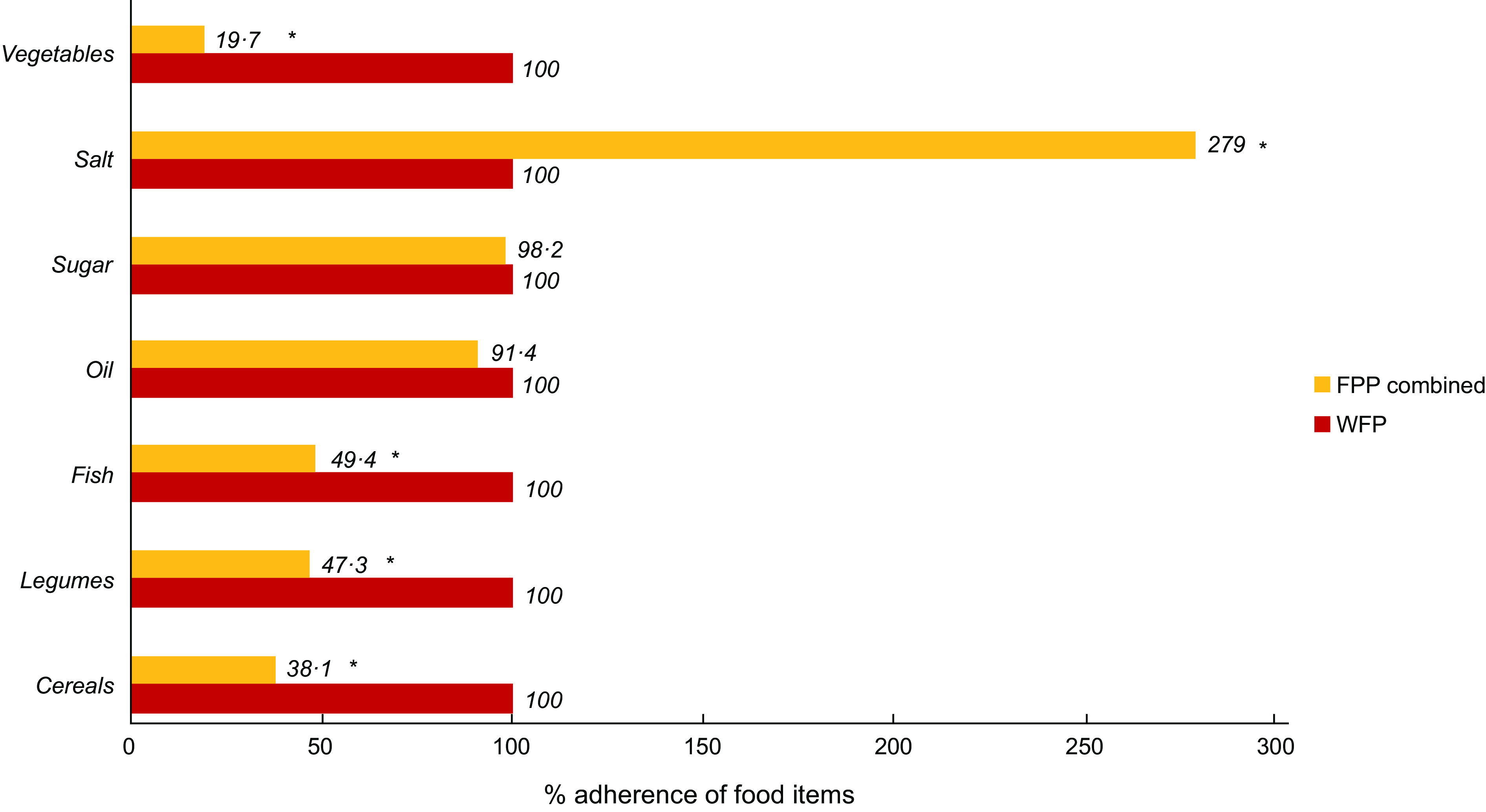
Percent of average adherence of all food parcel providers (FPP) compared with the locally tailored WFP guidelines for food parcel composition by food items†. *Significance *P*-value < 0.001. †Locally tailored WFP guidelines for food basket/parcel composition^(26)^. N of FPP combined = 72. WFP, World Food Programme

**Fig. 4:**
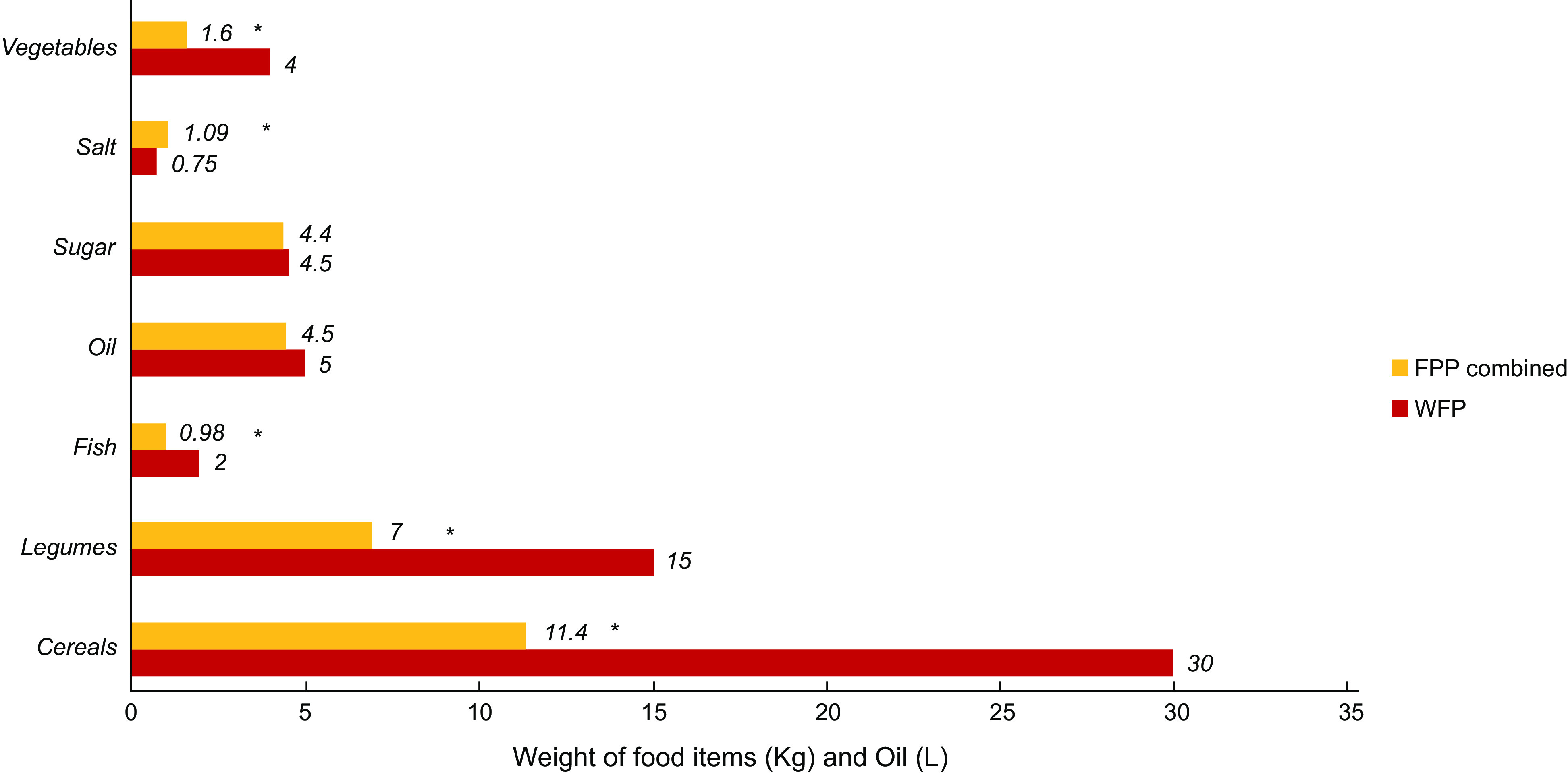
Comparison of the average weight of food parcel items provided by all food parcel providers (FPP) with the local WFP guidelines on food parcel composition†. *Significance P-Value < 0.001. †Locally tailored WFP guidelines for food basket/parcel composition^(26)^. N of FPPs combined = 72. WFP, World Food Programme

As shown in Fig. 3, the adherence of FPP included in the present study to the locally tailored WFP guidelines for the food parcel composition was found to be significantly low for specific food items, namely vegetables, fish, legumes and cereals (all *P*-values < 0·001). Results also showed that the recommended salt content of food parcels was significantly surpassed by all FPP (*P*-value < 0·001). On the other hand, the amounts (in kilograms) of salt and oil provided in the food parcels by FPP were in line with the WFP requirements for the food parcel items, and no statistical differences were noted (*P* > 0·05); see Fig. 4.

### Food basket nutrition quality and comparison to the dietary reference intakes (per person per d)

The estimated average energy and nutrient content of food parcels for a single person per d compared with the US DRI and the international WFP/FAO/WHO guidelines were presented in Table 2. Our results showed that on average, each food parcel provided 608 ± 55 kcal per person per d, which was significantly lower than the recommended energetic intakes, as per the WFP guidelines for dry food rations/food baskets (2100 kcal/d) and the US RDA for males and females (2725 kcal/d), *P* < 0·001. The greatest contributors to TE in the food parcel were carbohydrates (46 % TE ± 2) and fats (46 % TE ± 2), while protein contributed to 11 % TE (se: 0·9). Significant differences were noted between average nutrient content of food parcels and the recommended intakes for all nutrients per person per d (*P* < 0·001). Overall, energy provided in food parcels as carbohydrate was 71–103 % of the DRI, yet energy from sugar was 185 % of the recommended values. In addition, the food parcel content for fat was (276 % of the WFP guidelines (17 % of calories from fat) and between 134 and 234 % using the acceptable macronutrient distribution range by the US DRI. On the other hand, the dietary fibre content of food parcels helped meet only 15 % of daily needs. Scatterplots showing the contribution of food parcels to total daily energy and macronutrients (CHO, protein and fat) intake in comparison with standard DRI values were also presented (see online Supplemental material, Fig. S1). Results from Fig. S1 (*a–d)* further highlight the low contribution of food parcels to total daily energy needs (2725 kcal) and protein needs (46–56 g), yet the percent of energy from fat exceeded the DRI requirements (44–78 g).

**Table 2:** Estimated mean energy and nutrient content of food parcels from food parcel providers (*n* 72) in Lebanon, for a single person/d compared with the US dietary reference intakes and international guidelines

	Content of food parcel/person/d mean	se	Guidelines	% Needs met
Calories (kcal)	608	55	2100 kcal*	29¶
			2725 kcal‡	22¶
Macronutrients				
Carbohydrates (g)	64	4	130 g	49¶
Carbohydrate (% TE)	46	2	45–65†	71–103¶
Dietary fibre (g)	4·1	0·4	28 g‖	15¶
Sugars (g)	26	2		
Sugar (% TE)	19	1	< 10§	185¶
Fats (g)	35	5	44–78 g	45–79¶
Fat (% TE)	47	2	17*	276¶
Saturated fat (g)	5	0·6	20–35†	134–234¶
Saturated fat (% TE)	6	0·3	10§	62¶
*Trans*-fat (g)	0·07	0·01	<1§	10¶
Protein (g)	11	0·9	46–56 g	19¶
Protein (% TE)	7	0·3	10–12*	62–74¶
Micronutrients				
Vitamin A (mcg)	33	6	918‡	4¶
Vitamin D (mg)	0·5	0·1	18‡	3¶
Vitamin E (mg)	0·05	0·01	17‡	0·3¶
Vitamin K (mcg)	4	0·4	105‖	4¶
Vitamin C (mg)	1	0·2	93‡	2¶
Fe (mg)	2	0·2	16‡	12¶
Zn (mg)	1	0·1	11‡	13¶
Iodine (mg)	0·04	0·02	220‡	0·02¶
Na (mg)	183	28	1500‖	12¶
Potassium (mg)	266	28	2925‖	9¶
Ca (mg)	103	16	1100‡	9¶
Thiamin (mg)	0·1	0·01	1·3‡	11¶
Riboflavin (mg)	0·1	0·02	1·4‡	10¶
Niacin (mg)	1	0·1	16‡	8¶
B_6_ (mg)	0·2	0·01	1·7‡	9·3¶
Dietary folate (mcg)	59	5	500‡	12¶

% TE, percent total energy.

* World Food Programme international guidelines for food parcels based on the daily dietary needs set by the FAO/WHO^(24,25)^.

† Acceptable macronutrient distribution range^(28)^.

‡ Dietary guidelines for Americans 2020–2025^(45)^.

§ RDA have been averaged for males and females aged 19–75+^(28)^. The recommended energy allowances for adults with a light-to-moderate activity level were calculated by using the WHO (1985) equations^(27)^.

‖ Adequate intakes have been averaged for males and females aged 19–75+ years^(28)^.

¶ Nutrient content of food parcel differed significantly from the dietary guidelines using a one-sample *t* test (*P* <0·001).

In addition, the content of the parcel was significantly lower than the dietary guidelines for all micronutrients meeting only between 10 to 15 % of adults’ daily needs for Fe (12 %), Zn (13 %), thiamin (11 %), riboflavin (10 %) and folate (12 %), respectively. In addition, the food rations provide between 5 and 10 % of the recommended daily intakes for K (9 %), Ca (9 %), niacin (8 %) and vitamin B_6_ (9 %). Analysis showed that food rations include less than 5 % of the recommended daily intakes for several vitamins, including vitamins A, D, E, K and C (Table 2).

### Interest in attending trainings on food aid assistance

When asked if interested in attending training workshops to improve the food assistance content and adherence to the WFP or international guidelines, the majority of FPP (84·1 %, *n* 58) exhibited their interest. The remaining 16 % FPP who expressed their lack of interest or need for such trainings declared that they either were working with experienced food box distributors or have a licensed nutritionist/dietitian assisting with the food parcel content decisions. Additionally, 60 % (*n* 41) FPP did not express their interest in receiving nutrition tips and messages that can be distributed along with the food parcels to assist the beneficiaries in their food preparation of healthy and nutritious meals.

### Food safety measures adopted by food parcel providers

Thirty-seven (50·7 %) FPP filled the food items within their parcels in-house, while twenty-two (30·1 %) had them pre-packed and fourteen (19·2 %) had a mix of in-house and pre-packed parcels. When asked about checking expiry dates for food items within the parcels, 97 % of FPP confirmed that practice. In addition, 86 % of FPP reported having storage areas for their food parcels with proper ventilation. Concerning the COVID-19 safety measures adopted during food parcel packaging and distribution, 87 % FPP reported that all involved personnel and volunteers wore face masks and 89 % applied social distancing during the food parcel packaging and distribution processes. In addition, 80 % of FPP reported sanitising food items within each of the food parcels prior to their distribution.

### Impact of the crises on the prices of food boxes

FPP reported the costs of food parcels in both currencies, US dollars and Lebanese currency (LBP), given the recurrent changes in conversion rates during the economic crisis. Almost a quarter of FPP 22·7 % (*n* 15) reported buying their food items in USD dollars and 77·3 % (*n* 51) of FPP purchased food items in LBP currency.

The prices of the food boxes items changed during the distribution in both currencies with 73·5 % of FPP (*n* 50) reporting changes in cost of food parcels during their FPD. On average, the percent increase in price was 19·5 ± 69·638 in USD and 99·2 ± 84·781 in LBP. The average final price of food parcels at the time of data collection was reported as 36 ± 17 (USD) and 110 157 ± 63 991 (LBP). Additionally, 47·1 % (*n* 32) of the FPP reported changing food items included in the box to adjust to the recurrent changes in food item costs. More specifically, 19·1 % (*n* 13) of FPP reported changes only in the quantity of the items included within the food parcels in the basket, 14·7 % (*n* 10) reported changes only in quality and 11·8 % (*n* 8) reported changes in both the quantity and quality of food included in the parcel (see online Supplemental Material, Table 2).

## Discussion

Two years post the pandemic and the start of the financial crisis in the country, the mapping of the existing FPD, targeting primarily Lebanese households, remains limited at best. The present study is the first to our knowledge to assess the landscape of FPD in Lebanon and to explore the nutritional content and dietary adequacy of food parcels during the COVID-19 pandemic and an unprecedented economic crisis affecting the population’s food security. Our results show minimal adherence of FPP to the WFP recommendations for food parcels that were tailored for the local context. In addition, the nutritional content of food parcels was not in line with the dietary guidelines to meet daily needs of food parcel beneficiaries. Major challenges were also reported by the FPP with respect to food parcel costs and fluctuation in food prices, which may further affect the quantity and quality of food items included in the food parcels.

Our findings highlight heterogeneity in FPD among FPP in the present study, including variations in duration of distribution, selection criteria, geographical regions covered, sources of funds, and other forms of food and/or cash assistance provided to beneficiaries. The concentration of FPD was noted to be the highest in the capital Beirut with 60 % of FPP serving the city. This is not surprising given that the capital has the highest population density with 36 185 people residing per square kilometre compared to all other cities and villages across the country^(29)^. Worth noting that the number of FPP actively involved in FPD was observed to be the lowest in the governorates of Akkar and the Baalbeck-Hermel. These governorates are considered amongst the most underserved rural communities in the country with their populations experiencing heightened economic and social challenges coupled with food insecurity due to the compounded crises affecting the country^(24)^.

Results from the present study showed minimal adherence of FPP to the WFP food parcel composition guidelines that were tailored for the local context. On average, food parcels had lower than the recommended amounts of vegetables, legumes and fish compared to the requirements. In parallel, items included in the food parcels provided higher than the recommended levels of salt. Fruits distribution was not reported as part of the food parcels, mostly because dry rations usually lack fresh produce. Our results were in line with other studies conducted in high-income countries with food parcels distributed by food banks and charity-based organisations. In a study conducted on ninety-six parcels distributed by different food banks throughout the Netherlands, researchers showed that the dietary guidelines for a healthy diet were not met^(30)^. The food parcels provided in the same study had high energy content, with high SFA and proteins, yet very low fruits and vegetables, affecting the micronutrients requirements of recipients^(30)^. Similarly, a study assessing the quantity, quality and safety of the food parcels provided by programmes supplied by the Bread Food Bank in Toronto, Canada, indicated inadequate and low food quantity and poor nutritional quality as low in vitamins and minerals^(21)^. Likewise, a study done in Ontario displayed that food parcels distributed were inadequate and were not adhering to Canada’s Food Guide recommendations in terms of fruits and vegetables, meats and alternatives, and dairy products, with grains and cereals meeting the lower range of these recommendations^(31)^.

Findings from the present study also showed that the nutritional content of food parcels reflects their low contribution to total daily energy needs, yet the percent of energy from fat and sugar exceeded the DRI requirements. On the other hand, food parcels content had minimal contribution to daily needs for key micronutrients, including Fe, Zn, dietary folate, Ca, vitamin C and vitamin B complex. A recent mixed-method systematic review that investigated the nutritional adequacy of pre-packaged food parcels and their impact on the dietary intake of food bank users showed that food parcels distributed in several high-income countries, including the USA, UK, and Netherlands, were rather inconsistent at meeting nutritional requirements or individual needs, including cultural and health preferences^(32)^. Although the nutritional content of food parcels varied across and within studies within the same review, nutritional inadequacies that were consistently noted were those for fruits and vegetables as well as Fe and Ca. In addition, food parcels consistently exceeded energy requirements and provided energy-dense foods, which may contribute to poor diet quality amongst their beneficiaries^(32,33)^.

Several studies have also been conducted to date highlighting that food assistance, and the poor dietary quality of foods provided within food parcels, may contribute to exacerbation of health problems amongst beneficiaries. A study in Germany had previously highlighted the higher prevalence of diabetes and poorer health status among food bank beneficiaries compared with individuals of low socio-economic status from representative national surveys^(34)^. Similarly, high blood pressure, obesity, anaemia, folate and vitamin D deficiency were strongly predominant in unprivileged beneficiaries relying on food aid in France^(35)^. A recent study conducted by Basu *et al*.^(4)^ used micro-simulation exercises to assess the impact of various food aid modalities received by Palestinian refugees in the Middle East on their risk of chronic diseases. Their results showed that alternative food parcel options of higher quality (i.e. less grains and more of fruits and vegetables) can help decrease the incidence of hypertension, type 2 diabetes and CVD events more than electronic debit card delivery of food aid or cash transfers^(4)^. It is worth noting that in Lebanon, previous studies showed the overall low adherence of Lebanese youth and adults to the Mediterranean diet and the shift away from the traditional foods towards more westernised dietary patterns, especially among food-insecure households^(36–39)^. The nutrition transition witnessed in the country was also associated with increased levels of obesity and non-communicable diseases over the past two decades^(40,41)^. Thus, our study findings on the inadequate contribution of food parcels to the dietary intake of beneficiaries further highlight the need for food aid to be further scrutinised as it may contribute to poor diet quality and micronutrient deficiencies, thus exacerbating the problem of chronic nutrition-related diseases among food-insecure individuals.

Findings from the present study also showed that slightly above one-third of the FFP who completed the survey reported the inclusion of milk items (in powder or liquid form such as ultra-high temperature pasteurised milk) within their food parcels. These findings need to be considered with caution given the adoption of national and international guidelines on infant young child feeding practices (IYCF) in Lebanon, including Law 47 (issued in 2008) and the International Code of Marketing of Breast Milk Substitutes (‘Code’) that prohibit the distribution or promotion of formula or BMS. The practices documented in our study are also in violation of the local guidelines developed for the food parcel composition and that were adopted in the present study, as well as the IYCF Operational Guidance on infant feeding in emergencies and the SPHERE standards that necessitate the exclusion of infant formulas and other milk products as a separate commodity in the general FPD during emergency and non-emergency situations^(42,43)^. Similar violations have been previously reported in Lebanon through the distribution of milk in its different forms, including infant formulas, by donor agencies and/or local NGO to Lebanese households and Syrian refugees^(44)^. These findings further highlight the need for further enforcement of existing policies and programmes in Lebanon to support, promote and protect ICYF, particularly during the compounded crises witnessed in the country.

Most respondents from FPP in our study expressed their interest in attending educational trainings and workshops to improve their knowledge about food distribution systems. In terms of food safety and abidance by the COVID-19 protocols, all FPP representatives reported that guidelines have been well followed. These are promising findings and can be the basis to initiate capacity building and development for NGO dealing with food aid across the country.

Finally, a very important point to highlight from the present study is the continuous and worrying surge in prices, affecting the quality and content of food parcels, and even the very existence of food distribution initiatives. The biggest challenge reported by FPP were related to the devaluation of the local currency and the constant fluctuations in food prices, which affected FPP’s ability to secure food from suppliers and include the basic food items within their monthly food parcels. In summer 2020, during the data collection phase, the inflation rate was reported to be 89·7 %; however, with the ongoing economic crisis and the continuous devaluation of the currency, the rates of inflation are reaching unprecedented levels (162·47 %)^(45)^. The economic crisis witnessed in the country can threaten the food aid systems, their consistency and the quality of food items, while simultaneously increasing the beneficiaries’ reliance on food assistance. Furthermore, changing constantly the content of food parcels may threaten to further lower the nutritional quality of food items provided, which can adversely affect the diversity and adequacy of the diets consumed by beneficiaries.

### Strengths and limitations

Our results should be carefully interpreted in the context of the strengths and limitations of the study. One of the strengths of the study is the use of locally developed and tailored WFP guidelines to assess adherence to dietary guidelines for food parcels. In addition, the research team followed a rigorous methodology to receive detailed content description and pictures of the food parcels provided to beneficiaries while also receiving adequate training on conducting phone interviews. Nevertheless, the study has several limitations worth considering. The results from our study estimate the contribution of the food parcels provided by FPP to daily dietary intake of beneficiaries, but it does not take into consideration intra-household dynamics and household-level food allocation and distribution decisions. Such decisions can affect the amount of food consumed by beneficiaries from the food parcel or other sources, including women and children. In addition, our results do not take into consideration the contribution of supplementary sources of food aid and cash transfers that may affect the dietary intake and nutrient adequacy of beneficiaries. Thus, further analysis is needed to explore individual-level food intake and further determine the level of adequacy of dietary intake among beneficiaries receiving food aid from multiple sources and methods of humanitarian aid. Another limitation includes the use of the US-based DRI and international recommendations for adults given the lack of national dietary references to assess the nutrient adequacy of the food parcels. Albeit the use of the USDA food composition database, the research team expanded the database within the Nutri Pro software to include local food products and nutrient content from local brands. Another limitation of the study is its cross-sectional nature. Given the worsening of the economic situation in the country, particularly after the Beirut port blast that took place in August 2020 and the in the war in Ukraine that have had further repercussions on the food security situation at global and national levels, FPD and the quality of foods provided may have been further affected. Thus, follow-up studies are required to assess the dynamic and emergency nature of the situation. The need for rigorous and real-time tracking of data on the role of food aid and humanitarian responses in alleviating nutrition insecurity in conflict and crisis-affected countries remain evident gaps in the scientific and grey literatures.

## Conclusions

Our study confirms that the nutritional quality of food parcels supplied by various organisations and food distribution initiatives included in the analysis did not consistently adhere to the nutritional recommendations and food parcel guidelines. The improvements in food security and nutrition status during crises and emergencies necessitate careful monitoring of any food aid intervention, as well as an investment in long-term and more sustainable social protection programmes. In Lebanon, there is an acute need for better food aid planning and targeting. It is suggested that a national food security and nutrition policy should be developed, concentrating on relief operations while also considering longer-term policies and programmes that address the various determinants of optimal diets and nutrition. It is noteworthy to mention that in 2021, the Ministry of Public Health launched for the first time a National Nutrition Strategy and Action Plan for Lebanon (2021–2026) which has a goal to improve food security and ensure emergency preparedness, one way being through developing a national multi-sectoral policy framework and action plan to strengthen coordination among stakeholders^(46)^. Thus, under this new strategy, governmental agencies along with the active humanitarian sector can further streamline, coordinate and monitor the FPD by various entities in the country, including non-governmental charity- and religious-based organisations as well as personal initiatives. This can help limit the violations in distribution of infant formula and other milk substitutes while also improving the quality of foods provided within the parcels. Other improvements that can be considered in future food aid programming and distribution of food baskets is the promotion of fresh food through fresh fruits and vegetables vouchers, connecting with local farmer’s markets and community-based cooperatives, and mobilising the role of community and school gardens to enhance dietary diversity and quality of food consumed by beneficiaries. In conclusion, our findings highlight the need to improve the nutritional content of food parcels and adherence to dietary guidelines as part of the national strategies and initiatives that aim at alleviating food and nutrition insecurity in Lebanon while preventing diet-related diseases and adverse health outcomes amongst the most vulnerable.

## Supplementary material

For supplementary material accompanying this paper visit https://doi.org/10.1017/S1368980023000241. click here to view supplementary material
