# Supplementary material for: Assessing the nutritional content and adequacy of food parcels among vulnerable Lebanese during a double crisis: COVID-19 pandemic and an economic meltdown
Source: Public Health Nutr. 2023 Feb 13;26(6):1271–83. doi: 10.1017/S1368980023000241 (PMC10346028; doi:10.1017/S1368980023000241)
Supplement: Supplementary file 1 [file S1368980023000241sup.zip › S1368980023000241sup001.docx]

Supplementary material

Table S1. World Food Program Recommended guidelines

| Item | Recommendations for monthly Ration (Kg) |
| --- | --- |
| Cereals (including rice, pasta and bulgur) | 30 |
| Legumes (including lentils, chickpeas and beans) | 15 |
| Fish | 2 |
| Oil | 5 (Liter) |
| Vegetables (tomato paste and vegetables) | 4 |
| Salt | 0.75 |
| Sugar | 4.5 |
| Total | 61.25 |

Table S2: Impact of Price changes on the content of the food parcels

|  |  | Frequency | Percentage |
| --- | --- | --- | --- |
| Change items of the box | Yes | 32 | 47.1 |
|  | No | 36 | 52.9 |
| Changes in the box | Change in quantity | 13 | 19.1 |
|  | Change in quality | 10 | 14.7 |
|  | Change in quality and quantity | 8 | 11.8 |
|  | NA | 37 | 54.4 |
