# Supplementary material for: Assessing the nutritional content and adequacy of food parcels among vulnerable Lebanese during a double crisis: COVID-19 pandemic and an economic meltdown
Source: Public Health Nutr. 2023 Feb 13;26(6):1271–83. doi: 10.1017/S1368980023000241 (PMC10346028; doi:10.1017/S1368980023000241)

**Fig. 5 (a-d)** Scatter plot graphs showing contribution of food parcels to the estimated todal energy intake and macronutrients to standard DRI values.


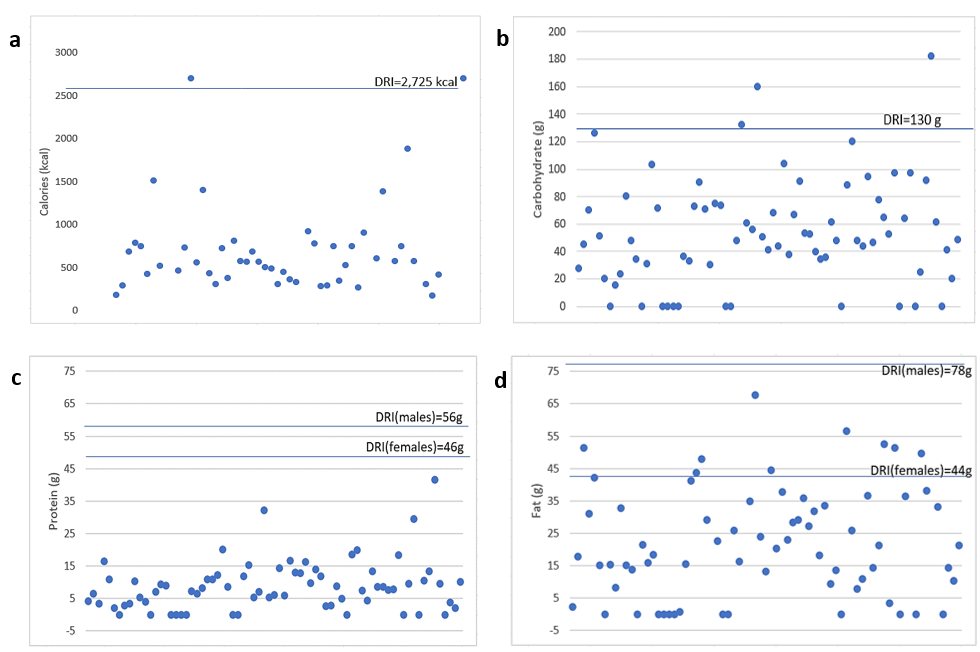

Supplement: Supplementary file 1 [file S1368980023000241sup.zip › S1368980023000241sup002.docx]
